# Computing Classical-Quantum Channel Capacity Using Blahut–Arimoto Type Algorithm: A Theoretical and Numerical Analysis †

**DOI:** 10.3390/e22020222

**Published:** 2020-02-16

**Authors:** Haobo Li, Ning Cai

**Affiliations:** 1 School of Information Science and Technology, ShanghaiTech University, Shanghai 201210, China; 2 Shanghai Institute of Microsystem and Information Technology, Chinese Academy of Sciences, Shanghai 201210, China; 3 University of Chinese Academy of Sciences, Beijing 100049, China

**Keywords:** capacity, classical-quantum channel, Blahut–Arimoto type algorithm, convergence speed

## Abstract

Based on Arimoto’s work in 1972, we propose an iterative algorithm for computing the capacity of a discrete memoryless classical-quantum channel with a finite input alphabet and a finite dimensional output, which we call the Blahut–Arimoto algorithm for classical-quantum channel, and an input cost constraint is considered. We show that, to reach ε accuracy, the iteration complexity of the algorithm is upper bounded by $\frac{\log n\log\varepsilon }{\varepsilon }$ where *n* is the size of the input alphabet. In particular, when the output state ${\left\{\rho_{x}\right\}}_{x\in X}$ is linearly independent in complex matrix space, the algorithm has a geometric convergence. We also show that the algorithm reaches an ε accurate solution with a complexity of $O(\frac{m^{3}\log n\log\varepsilon }{\varepsilon })$, and $O(m^{3}\log\varepsilon \log_{\left(1-\delta \right)}\frac{\varepsilon }{D(p^{*}||p^{N_{0}})})$ in the special case, where *m* is the output dimension, $D(p^{*}||p^{N_{0}})$ is the relative entropy of two distributions, and δ is a positive number. Numerical experiments were performed and an approximate solution for the binary two-dimensional case was analysed.

## 1. Introduction

The computation of channel capacity has always been a core problem in information theory. The very well-known Blahut-Arimoto algorithm [1,2] was proposed in 1972 to compute the discrete memoryless classical channel. Inspired by this algorithm, we propose an algorithm of Blahut-Arimoto type to compute the capacity of discrete memoryless classical-quantum channel. The classical-quantum channel [3] can be considered as a mapping $x\to \rho_{x}$ of an input alphabet X={1,2,⋯,|X|} to a set of quantum states in a finite dimensional Hilbert space H. The state of a quantum system is given by a density operator ρ, which is a positive semi-definite operator with trace equal to one. Let $D^{m}$ denote the set of all density operators acting on a Hilbert space H of dimension *m*. If the source emits a letter *x* with probability $p_{x}$, the output would be $\rho_{x}$, thus the output would form an ensemble: ${\left\{p_{x}:\rho_{x}\right\}}_{x\in X}$.

In 1998, Holevo showed [4] that the classical capacity of the classical-quantum channel is the maximization of a quantity called the Holevo information over all input distributions. The Holevo information χ of an ensemble ${\left\{p_{x}:\rho_{x}\right\}}_{x\in X}$ is defined as

(1) $$\begin{array}{c} \chi \left({\left\{p_{x}:\rho_{x}\right\}}_{x\in X}\right)=H\left(\sum_{x}p_{x}\rho_{x}\right)-\sum_{x}p_{x}H\left(\rho_{x}\right), \end{array}$$

where H(·) is the von Neumann entropy, which is defined on positive semidefinite matrices:

(2) $$\begin{array}{c} H(\rho )=-\mathrm{Tr}(\rho \log\rho ). \end{array}$$

Due to the concavity of von Neumann entropy [5], the Holevo information is always non-negative. The Holevo quantity is concave in the input distribution [5], and thus the maximization of Equation (1) over *p* is a convex optimization problem. However, it is not a straightforward convex optimization problem. In 2014, Sutter et al. [6] promoted an algorithm based on duality of convex programming and smoothing techniques [7] with a complexity of $O(\frac{\left(n\vee m\right)m^{3}{\left(\log n\right)}^{1/2}}{\varepsilon })$, where n∨m=max{n,m}.

For discrete memoryless classical channels, the capacity can be computed efficiently by using an algorithm called Blahut–Arimoto (BA) algorithm [1,2,8]. In 1998, H. Nagaoka [9] proposed a quantum version of BA algorithm. In his work, he considered the quantum-quantum channel and this problem was proved to be NP-complete in 2008 [10]. Despite the NP-completeness, in [11], an example is given of a qubit quantum channel which requires four inputs to maximize the Holevo capacity. Further research of Nagaoka’s algorithm was presented in [12], where the algorithm was implemented to check the additivity of quantum channels. In [9], Nagaoka mentioned an algorithm concerning classical-quantum channel; however, its speed of convergence was not studied there and the details of the proof were not presented either. In this paper, we show that, with proper manipulations, the BA algorithm can be applied to computing the capacity of classical-quantum channel with an input constraint efficiently. The remainder of this article is structured as follows. In Section 2, we propose the algorithm and show how the algorithm works. In Section 3, we provide the convergence analysis of the algorithm. In Section 4, we show the numerical experiments of BA algorithm to see how well this algorithm performs. In Section 5, we propose an approximate solution for a special case, which is the binary input, two-dimensional output case.

**Notations**: The logarithm with basis 2 is denoted by log(·). The space of all Hermitian operators of dimension *m* is denoted by $H^{m}$. The set of all density matrices of dimension *m* is denoted by $D^{m}\left\{\rho \in H^{m}:\rho \geq 0,\mathrm{Tr}\rho =1\right\}$. Each letter x∈X is mapped to a density matrix $\rho_{x}$, thus the classical-quantum channel can be represented as a set of density matrices ${\left\{\rho_{x}\right\}}_{x\in X}$. The set of all probability distributions of length *n* is denoted by $\Delta_{n}\left\{p\in R^{n}:p_{x}\geq 0,\sum_{x=1}^{n}p_{x}=1\right\}$. The von Neumann entropy of a density matrix ρ is denoted by H(ρ)=−Tr[ρlogρ]. The relative entropy between $p,q\in \Delta_{n}$, if supp(p)⊂supp(q), is denoted by $D\left(p||q\right)=\sum_{x}p_{x}\left(\log p_{x}-\log q_{x}\right)$ and +∞ otherwise. The relative entropy between $\rho ,\sigma \in D^{m}$, if supp(ρ)⊂supp(σ), is denoted by D(ρ||σ)=Tr[ρ(logρ−logσ)] and +∞ otherwise.

## 2. Blahut–Arimoto Algorithm for Classical-Quantum Channel

First, we write down the primal optimization problem:

(3) $$\begin{array}{c} \mathrm{Primal}:\left\{\begin{array}{c} \max_{p}H\left(\sum_{x}p_{x}\rho_{x}\right)-\sum_{x}p_{x}H\left(\rho_{x}\right),\\ \;\mathrm{subject}\;\mathrm{to}\;\;s^{T}p\leq S;\\ \;\;p\in \Delta_{n}, \end{array}\right. \end{array}$$

where $\rho_{x}\in D^{m}$, $s\in R^{n}$ is a positive real vector, S>0. We denote the maximal value of Equation (3) as C(S). In this optimization problem, we are to maximize the Holevo quantity with respect to the input distribution ${\left\{p_{x}\right\}}_{x\in X}$. Practically, the preparation of different signal state *x* has different cost, which is represented by $s=(s_{1},s_{2},\cdots ,s_{n})$. We would like to bound the expected cost of the resource within some quantity, which is represented by the inequality constraint in Equation (3).

**Lemma** **1.** *[6] Let a set G be defined as $G:=\arg\max_{p\in \Delta_{n}}\chi \left({\left\{p_{x}:\rho_{x}\right\}}_{x\in X}\right)$ and $S_{max}:=\min_{p\in G}s^{T}p$. Then, if $S\geq S_{max}$, the inequality constraint in the primal problem is inactive; and, if $S<S_{max}$, the inequality constraint in the primal problem is equivalent to $s^{T}p=S$.*

Now, we assume that $\min{\left\{s_{x}\right\}}_{x\in X}\leq S\leq S_{max}$. The Lagrange dual problem of Equation (3) is

(4) $$\begin{array}{c} \mathrm{Dual}:\left\{\begin{array}{cc} \; & \min_{\lambda \geq 0}\max_{p}H\left(\sum_{x}p_{x}\rho_{x}\right)-\sum_{x}p_{x}H\left(\rho_{x}\right)-\lambda \left(s^{T}p-S\right)\\ \; & \mathrm{subject}\;\mathrm{to}\;p\in \Delta_{n}. \end{array}\right. \end{array}$$

**Lemma** **2.** *Strong duality holds between Equations (3) and (4).*

**Proof.**  The lemma follows from standard strong duality result of convex optimization theory ([13], Chapter 5.2.3). □

Define functions. Let

(5) $$\begin{array}{cc} f_{\lambda }\left(p,p^{\prime }\right) & =\sum_{x}\mathrm{Tr}\left\{p_{x}\rho_{x}\left[\log\left(p_{x}^{\prime }\rho_{x}\right)-\log\left(p_{x}\rho^{\prime }\right)\right]\right\}-\lambda s^{T}p, \end{array}$$

(6) $$\begin{array}{cc} F(\lambda ) & =\max_{p}\max_{p^{\prime }}f\left(p,p^{\prime }\right). \end{array}$$

where $\rho^{\prime }=\sum_{x}p_{x}^{\prime }\rho_{x}$.

**Lemma** **3.** *For fixed p, $\arg\max_{p^{\prime }}f_{\lambda }\left(p,p^{\prime }\right)=p$.*

**Proof.**  Actually, we can prove a stronger lemma (the following lemma was proposed in [9], but no proof was given, perhaps due to the space limitation). We now restate the lemma in [9] and give the proof. □

**Lemma** **4.** *For fixed ${\left\{p_{x}:\rho_{x}\right\}}_{x\in X}$, we have*

(7) $$\begin{array}{c} \max_{{\left\{q_{x}:\sigma_{x}\right\}}_{x\in X}}-D\left(p||q\right)+\sum_{x}p_{x}\mathrm{Tr}\left\{\rho_{x}\left[\log\sigma_{x}-\log\sigma \right]\right\}=\sum_{x}p_{x}\mathrm{Tr}\left\{\rho_{x}\left[\log\rho_{x}-\log\rho \right]\right\},\\ i.e.,\;\arg\max_{{\left\{q_{x}:\sigma_{x}\right\}}_{x\in X}}-D\left(p||q\right)+\sum_{x}p_{x}\mathrm{Tr}\left\{\rho_{x}\left[\log\sigma_{x}-\log\sigma \right]\right\}\;={\left\{p_{x}:\rho_{x}\right\}}_{x\in X}, \end{array}$$

*where $p,q\in \Delta_{n},\sigma_{x}\in D^{m}$ and $\rho =\sum_{x}p_{x}\rho_{x},\;\sigma =\sum_{x}q_{x}\sigma_{x}$.*

**Proof.**  Considering Equation (7), we have

$$\begin{array}{cc} RHS-LHS & =D\left(p||q\right)+\sum_{x}p_{x}D(\rho_{x}\left|\right|\sigma_{x})-D(\rho ||\sigma )\\ & =D(\rho_{XB}\left|\right|\sigma_{XB})-D(\rho ||\sigma ), \end{array}$$

where $\rho_{XB}=\sum_{x}p_{x}{\left|x\rangle \langle x\right|}_{X}⊗\rho_{x}$ and $\sigma_{XB}=\sum_{x}q_{x}{\left|x\rangle \langle x\right|}_{X}⊗\sigma_{x}$ are classical-quantum state [5]. Let the quantum channel N be the partial trace channel on *X* system; then, by the monotonicity of quantum relative entropy ([5], Theorem 11.8.1), we have

$$\begin{array}{c} D(\rho_{XB}\left|\right|\sigma_{XB})\geq D(N\left(\rho_{XB}\right)||N\left(\sigma_{XB}\right))=D(\rho ||\sigma ). \end{array}$$

□

Notice that, if we let $\sigma_{x}=\rho_{x}$ in Equation (7), with some calculation, Equation (7) becomes Lemma 3. Thus, Lemma 3 is a straightforward corollary of Lemma 4

**Theorem** **1.** *The dual problem in Equation (4) is equivalent to*

(8) $$\begin{array}{c} \min_{\lambda \geq 0}F\left(\lambda \right)+\lambda S. \end{array}$$

**Proof.**  It follows from Equation (5) and Lemma 3 that

$$\begin{array}{cc} \; & \max_{p^{\prime }}f_{\lambda }\left(p,p^{\prime }\right)=f_{\lambda }\left(p,p\right)=H\left(\rho \right)-\sum_{x}p_{x}H\left(\rho_{x}\right)-\lambda s^{T}p. \end{array}$$

Hence,

$$\begin{array}{cc} \; & \min_{\lambda \geq 0}\max_{p}H\left(\rho \right)-\sum_{x}p_{x}H\left(\rho_{x}\right)-\lambda \left(s^{T}p-S\right)\\ = & \min_{\lambda \geq 0}\max_{p}\max_{p^{\prime }}f_{\lambda }\left(p,p^{\prime }\right)+\lambda S\\ = & \min_{\lambda \geq 0}F\left(\lambda \right)+\lambda S. \end{array}$$

□

The BA algorithm is an alternating optimization algorithm, i.e., to optimize $f_{\lambda }\left(p,p^{\prime }\right)$, each iteration step would fix one variable and optimize the function over the other variable. Now, we use BA algorithm to find F(λ). The iteration procedure is

$$\begin{array}{cc} \; & p_{x}^{0}>0,\\ \; & p_{x}^{{}^{\prime }t}=p_{x}^{t}\;,\\ \; & p^{t+1}=\arg\max_{p}\sum_{x}\mathrm{Tr}\left\{p_{x}\rho_{x}\left[\log\left(p_{x}^{t}\rho_{x}\right)-\log\left(p_{x}\rho^{t}\right)\right]\right\}-\lambda s^{T}p, \end{array}$$

where $\rho^{t}=\sum_{x}p_{x}^{t}\rho_{x}$.

To get $p^{t+1}$, we can use the Lagrange function:

$$\begin{array}{cc} L= & \sum_{x}\mathrm{Tr}\left\{p_{x}\rho_{x}\left[\log\left(p_{x}^{t}\rho_{x}\right)-\log\left(p_{x}\rho^{t}\right)\right]\right\}-\lambda s^{T}p-\nu \left(\sum_{x}p_{x}-1\right), \end{array}$$

setting the gradient with respect to $p_{x}$ to zero. By combining the normalization condition, we can have (taking the natural logarithm for convenience):

(9) $$\begin{array}{cc} p_{x}^{t+1}= & \frac{r_{x}^{t}}{\sum_{x}r_{x}^{t}}\;, \end{array}$$

(10) $$\begin{array}{cc} \mathrm{where}\;\;\;r_{x}^{t}= & \exp\left(\mathrm{Tr}\left\{\rho_{x}\left[\log\left(p_{x}^{t}\rho_{x}\right)-\log\rho^{t}\right]\right\}-s_{x}\lambda \right),\\ \rho^{t}= & \sum_{x}p_{x}^{t}\rho_{x}. \end{array}$$

Thus, we can summarize the Algorithm 1 below.

**Algorithm 1** Blahut–Arimoto algorithm for discrete memoryless classical-quantum channel.
set $p_{x}^{0}=\frac{1}{\left|X\right|}$, x∈X;
**repeat**
    $p_{x}^{{}^{\prime }t}=p_{x}^{t};$    $p_{x}^{t+1}=\frac{r_{x}^{t}}{\sum_{x}r_{x}^{t}}$, where $r_{x}^{t}=\exp\left(\mathrm{Tr}\left\{\rho_{x}\left[\log\left(p_{x}^{t}\rho_{x}\right)-\log\rho^{t}\right]\right\}-s_{x}\lambda \right)$;   **until** convergence.

**Lemma** **5.** Let $p^{*}\left(\lambda \right)=\arg\max_{p}f\left(p,p\right)$ for a given λ; then, $s^{T}p^{*}\left(\lambda \right)$ is a decreasing function of λ.

**Proof.**  For convenience, we denote $\chi ({\left\{p_{x}:\rho_{x}\right\}}_{x\in X})$ as χ(p). Notice that $f_{\lambda }\left(p,p\right)=\chi \left(p\right)-\lambda s^{T}p$ by definition of f(p,p).

For $\lambda_{1}<\lambda_{2}$, if $\langle p^{*}\left(\lambda_{1}\right)s<s^{T}p^{*}\left(\lambda_{2}\right)$, then, by the definition of $p^{*}\left(\lambda \right)$, we have:

$$\begin{array}{cc} \chi \left(p^{*}\left(\lambda_{1}\right)\right)-\lambda_{1}s^{T}p^{*}\left(\lambda_{1}\right)\geq & \chi \left(p^{*}\left(\lambda_{2}\right)\right)-\lambda_{1}s^{T}p^{*}\left(\lambda_{2}\right)\\ ⟹\chi \left(p^{*}\left(\lambda_{2}\right)\right)-\chi \left(p^{*}\left(\lambda_{1}\right)\right)\leq & \lambda_{1}s^{T}\left(p^{*}\left(\lambda_{2}\right)-p^{*}\left(\lambda_{1}\right)\right)\\ < & \lambda_{2}s^{T}\left(p^{*}\left(\lambda_{2}\right)-p^{*}\left(\lambda_{1}\right)\right)\\ ⟹\chi \left(p^{*}\left(\lambda_{1}\right)\right)-\lambda_{2}s^{T}p^{*}\left(\lambda_{1}\right)> & \chi \left(p^{*}\left(\lambda_{2}\right)\right)-\lambda_{2}s^{T}p^{*}\left(\lambda_{2}\right), \end{array}$$

which is a contradiction to the fact that $p^{*}\left(\lambda_{2}\right)$ is an optimizer of $\chi \left(p\right)-\lambda_{2}s^{T}p$. Thus, we must have $s^{T}p^{*}\left(\lambda_{1}\right)\geq s^{T}p^{*}\left(\lambda_{2}\right)$ if $\lambda_{1}<\lambda_{2}$. □

We do not need to solve the optimization problem in Equation (8), because from Lemma 1 we can see that the statement “$p^{*}$ is an optimal solution” is equivalent to “$s^{T}p^{*}=S$ and $p^{*}$ maximizes $f_{\lambda }\left(p,p\right)+\lambda S=\chi \left({\left\{p_{x},\rho_{x}\right\}}_{x\in X}\right)-\lambda \left(s^{T}p-S\right)$”, which is also equivalent to “$s^{T}p^{*}=S$ and $p^{*}$ maximizes $f_{\lambda }\left(p,p\right)$", thus, if for some λ≥0, a *p* maximizes $f_{\lambda }\left(p,p\right)$ and $s^{T}p=S$, then the capacity C(S)=F(λ)+λS, and such λ is easy to find since $s^{T}p$ is a decreasing function of λ, and, to reach an ε accuracy, we need

(11) $$\begin{array}{c} O(\log\varepsilon ) \end{array}$$

steps using bisection method.

## 3. Convergence Analysis

Next, we show that the algorithm indeed converges to F(λ) and then provide an analysis of the speed of the convergence.

### 3.1. The Convergence Is Guaranteed

**Corollary** **1.** $$\begin{array}{c} f_{\lambda }\left(p^{t+1},p^{t}\right)=\log\left(\sum_{x}r_{x}^{t}\right). \end{array}$$

**Proof.**  $$\begin{array}{cc} f_{\lambda }\left(p^{t+1},p^{t}\right)= & -\sum_{x}\mathrm{Tr}\left\{p_{x}^{t+1}\rho_{x}\log p_{x}^{t+1}\right\}+\sum_{x}\mathrm{Tr}\left\{p_{x}^{t+1}\rho_{x}\left[\log\left(p_{x}^{t}\rho_{x}\right)-\log\left(\rho^{t}\right)\right]\right\}-\lambda s^{T}p^{t+1}\\ = & -\sum_{x}p_{x}^{t+1}\log p_{x}^{t+1}+\sum_{x}p_{x}^{t+1}\log\left(r_{x}^{t}\right)\\ = & \sum_{x}p_{x}^{t+1}\log\left(\frac{r_{x}^{t}}{p_{x}^{t+1}}\right)\\ = & \log(\sum_{x}r_{x}^{t}). \end{array}$$

The first equality comes from a manipulation of Equation (5). The second equality follows from Equation (10). The last equality follows from Equation (9). □

**Corollary** **2.** *For arbitrary distribution ${\left\{p_{x}\right\}}_{x\in X}$, we have*

$$\begin{array}{c} \chi \left({\left\{p_{x},\rho_{x}\right\}}_{x\in X}\right)-\lambda s^{T}p-f\left(p^{t+1},p^{t}\right)\leq \sum_{x}p_{x}\log\left(\frac{p_{x}^{t+1}}{p_{x}^{t}\left(x\right)}\right). \end{array}$$

**Proof.**  Define $\rho =\sum_{x}p_{x}\rho_{x}$. Then, we have

(12) $$\begin{array}{cc} \; & \sum_{x}p_{x}\log\left(\frac{p_{x}^{t+1}}{p_{x}^{t}}\right)=\sum_{x}p_{x}\log\left(\frac{1}{p_{x}^{t}}\frac{r_{x}^{t}}{\sum_{x^{\prime }}r_{x^{\prime }}^{t}}\right)\\ = & -f_{\lambda }\left(p^{t+1},p^{t}\right)+\sum_{x}p_{x}\log\frac{r_{x}^{t}}{p_{x}^{t}}\\ = & -f_{\lambda }\left(p^{t+1},p^{t}\right)+\sum_{x}p_{x}\mathrm{Tr}\left\{\rho_{x}\left[\log\left(p_{x}^{t}\rho_{x}\right)-\log\rho^{t}\right]-s_{x}\lambda -\log p_{x}^{t}\right\}\\ = & -f_{\lambda }\left(p^{t+1},p^{t}\right)+\sum_{x}p_{x}\mathrm{Tr}\left\{\rho_{x}\left[\log\rho_{x}-\log\rho^{t}\right]\right\}-\lambda s^{T}p\\ = & -f_{\lambda }\left(p^{t+1},p^{t}\right)+\sum_{x}p_{x}\mathrm{Tr}\left\{\rho_{x}\left[\log\rho_{x}-\log\rho +\log\rho -\log\rho^{t}\right]\right\}-\lambda s^{T}p\\ = & -f_{\lambda }\left(p^{t+1},p^{t}\right)+\chi \left({\left\{p_{x},\rho_{x}\right\}}_{x\in X}\right)-\lambda s^{T}p+D(\rho ||\rho^{t}). \end{array}$$

The first equality follows from Equation (9). The second equality follows from Corollary 1. The third equality follows from Equation (10). The last equality follows from Equation (1). Since the relative entropy $D(\rho^{X}||\rho^{t})$ is always non-negative [5], we have

$$\begin{array}{c} \chi \left({\left\{p_{x},\rho_{x}\right\}}_{x\in X}\right)-\lambda s^{T}p-f_{\lambda }\left(p^{t+1},p^{t}\right)\leq \sum_{x}p_{x}\log\left(\frac{p_{x}^{t+1}}{p_{x}^{t}\left(x\right)}\right). \end{array}$$

□

**Theorem** **2.** *$f_{\lambda }\left(p^{t+1},p^{t}\right)$ converges to F(λ) as t→∞.*

**Proof.**  Let $p^{*}$ be an optimal solution that achieves F(λ); then, we have the following inequality

(13) $$\begin{array}{cc} \; & \sum_{t=0}^{N}\left[F\left(\lambda \right)-f_{\lambda }\left(p^{t+1},p^{t}\right)\right]\\ = & \sum_{t=0}^{N}\left[\chi \left({\left\{p_{x}^{*},\rho_{x}\right\}}_{x\in X}\right)-\lambda s^{T}p^{*}-f_{\lambda }\left(p^{t+1},p^{t}\right)\right]\\ \leq & \sum_{t=0}^{N}\sum_{x}p_{x}^{*}\log\left(\frac{p_{x}^{t+1}}{p_{x}^{t}}\right)\\ = & \sum_{x}p_{x}^{*}\sum_{t=0}^{N}\log\left(\frac{p_{x}^{t+1}}{p_{x}^{t}}\right)\\ = & \sum_{x}p_{x}^{*}\log\left(\frac{p_{x}^{N+1}}{p_{x}^{0}}\right)\\ = & \sum_{x}p_{x}^{*}\log\left(\frac{p_{x}^{*}}{p_{x}^{0}}\right)+\sum_{x}p_{x}^{*}\log\left(\frac{p_{x}^{N+1}}{p^{*}\left(x\right)}\right)\\ = & D(p^{*}\left|\right|p^{0})-D(p^{*}\left|\right|p^{N+1}) \end{array}$$

(14) $$\begin{array}{c} \leq D(p^{*}||p^{0}). \end{array}$$

The first equality follows from Equations (5), (6), and (1). The first inequality follows from Corollary 2. The last inequality follows from the non-negativity of relative entropy.

Thus, let N→∞ and with $F\left(\lambda \right)-f_{\lambda }\left(p^{t+1},p^{t}\right)\geq 0$, we have

(15) $$\begin{array}{c} 0\leq \sum_{t=0}^{\infty }\left[F\left(\lambda \right)-f_{\lambda }\left(p^{t+1},p^{t}\right)\right]\leq D(p^{*}\left|\right|p^{0}), \end{array}$$

Notice we take the initial $p^{0}$ to be uniform distribution, so the right hand side of Equation (15) is finite. Combine with the fact that $f_{\lambda }\left(p^{t+1},p^{t}\right)$ is a non-decreasing sequence, this means $f_{\lambda }\left(p^{t+1},p^{t}\right)$ converges to F(λ). □

**Theorem** **3.** *The probability distribution ${\left\{p^{t}\right\}}_{t=0}^{\infty }$ also converges.*

**Proof.**  Remove the summation over *t* in Equations (13) and (14); then, we have

(16) $$\begin{array}{c} 0\leq F\left(\lambda \right)-f_{\lambda }\left(p^{t+1},p^{t}\right)\leq \sum_{x}p_{x}^{*}\log\left(\frac{p_{x}^{t+1}}{p_{x}^{t}}\right)=D(p^{*}\left|\right|p^{t})-D(p^{*}\left|\right|p^{t+1}). \end{array}$$

Now that the sequence ${\left\{p^{t}\right\}}_{t=0}^{\infty }$ is a bounded sequence, there exists a subsequence ${\left\{p^{t_{k}}\right\}}_{k=0}^{\infty }$ that converges. Let us say it converges to $\bar{p}$. Then, clearly, we have $f\left(\bar{p},\bar{p}\right)=F\left(\lambda \right)$ (or $f(p^{t+1},p^{t})$ would not converge). Substituting $p^{*}=\bar{p}$ in Equation (16), we have

$$\begin{array}{c} 0\leq D(\bar{p}\left|\right|p^{t})-D(\bar{p}\left|\right|p^{t+1}). \end{array}$$

Thus, the sequence $\{D(\bar{p}\left|\right|p^{t}{)\}}_{t=0}^{\infty }$ is a decreasing sequence and there exists a subsequence $\{D(\bar{p}\left|\right|p^{t_{k}}{)\}}_{k=0}^{\infty }$ that converges to zero. Therefore, we can conclude that $\{D(\bar{p}\left|\right|p^{t}{)\}}_{t=0}^{\infty }$ converges to zero, which means ${\left\{p^{t}\right\}}_{t=0}^{\infty }$ converges to $\bar{p}$. □

### 3.2. The Speed of Convergence

**Theorem** **4.** *To reach ε accuracy to F(λ), the algorithm needs an iteration complexity less than $\frac{\log n}{\varepsilon }$.*

**Proof.**  From the proof of Theorem 2, we know

$$\begin{array}{cc} \; & \sum_{t=0}^{N}\left[F\left(\lambda \right)-f_{\lambda }\left(p^{t+1},p^{t}\right)\right]\leq D(p^{*}\left|\right|p^{0})\\ = & \sum_{x}p_{x}^{*}\log\left(\frac{p_{x}^{*}}{p_{x}^{0}}\right)=\log n-H\left(p^{*}\right)<\log n, \end{array}$$

and $\left[F\left(\lambda \right)-f_{\lambda }\left(p^{t+1},p^{t}\right)\right]$ is non-increasing in *t*, thus

$$\begin{array}{c} F\left(\lambda \right)-f_{\lambda }\left(p^{t+1},p^{t}\right)<\frac{\log n}{t}. \end{array}$$

□

Next, we show that in some special cases the algorithm has a better convergence performance, which is a geometric speed of convergence.

**Assumption** **1.** *The channel matrices ${\left\{\rho_{x}\right\}}_{x\in X}$ are linearly independent, i.e., there does not exist a vector $c\in R^{n}$ such that*

$$\begin{array}{c} \sum_{x}c_{x}\rho_{x}=0. \end{array}$$

**Remark** **1.** *Assumption 1 is equivalent to: The output state $\rho =\sum_{x}p_{x}\rho_{x}$ is uniquely determined by the input distribution p.*

**Theorem** **5.** Under Assumption 1, the optimal solution $p^{*}$ is unique.

**Proof.**  Notice that the von Neumann entropy in Equation (2) is strictly concave [14], thus, for distributions $p\neq p^{\prime }$, $\rho =\sum_{x}p_{x}\rho_{x}\neq \sum_{x}p_{x}^{\prime }\rho_{x}=\rho^{\prime }$, which is followed from Assumption refas1. Thus, this means H(ρ) is strictly concave in *p*. Thus, Holevo quantity in Equation (1) is strictly concave in *p*, which means the optimal solution $p^{*}$ is unique. □

We also need the following theorem:

**Theorem** **6.** *[15] The relative entropy satisfies*

$$\begin{array}{c} D\left(\rho ||\sigma \right)\geq \frac{1}{2}\mathrm{Tr}{\left(\rho -\sigma \right)}^{2}. \end{array}$$

Now, we state the theorem of convergence:

**Theorem** **7.** Suppose start from some initial point $p^{0}$, then, under Assumption 1, the algorithm converges to the optimal point $p^{*}$, and $p^{0}$ converges to $p^{*}$ at a geometric speed, i.e., there exist $N_{0}$ and δ>0, where N and δ are independent, such that, for any $t>N_{0}$, we have

$$\begin{array}{c} D(p^{*}\left|\right|p^{t}{)\leq \left(1-\delta \right)}^{t-N_{0}}D(p^{*}\left|\right|p^{N_{0}}). \end{array}$$

**Proof.**  Define $d_{x}=p_{x}^{*}-p_{x}^{t}$ and the real vector $|d\rangle ={\left(d_{1},d_{2},\cdots ,d_{n}\right)}^{T}$. Using Taylor expansion, we have

$$\begin{array}{cc} D(p^{*}||p^{t})= & \sum_{x}p_{x}^{*}\log\left(\frac{p_{x}^{*}}{p_{x}^{t}}\right)=\sum_{x}-p_{x}^{*}\log\left(1-\frac{d_{x}}{p_{x}^{*}}\right)\\ = & \frac{1}{2}\langle d|P|d\rangle +\sum_{x}O\left(d_{x}^{3}\right), \end{array}$$

where $P=diag(p_{1}^{*},p_{2}^{*},\cdots ,p_{n}^{*})$. Now, $p^{t}$ converges to $p^{*}$, i.e., |d〉 converges to zero, thus there exists a $N_{0}$ such that, for any $t>N_{0}$, we have

(17) $$\begin{array}{c} D(p^{*}\left|\right|p^{t})\leq \frac{2}{3}\langle d|P|d\rangle . \end{array}$$

From Theorem 6, we have

(18) $$\begin{array}{c} D(\rho^{*}\left|\right|\rho^{t})\geq \frac{1}{2}\mathrm{Tr}\left\{{\left[\sum_{x}d_{x}\rho_{x}\right]}^{2}\right\}=\frac{1}{2}\langle d|M|d\rangle , \end{array}$$

where $M\in R^{n\times n}$:

$$\begin{array}{c} M_{ij}=\mathrm{Tr}\left(\rho_{i}\rho_{j}\right). \end{array}$$

From Equation (18), we know that, under Assumption 1, *M* is positive definite. Thus, there exists a δ>0 such that

$$\begin{array}{cc} \; & \frac{1}{2}M>\delta \frac{2}{3}P\Rightarrow \frac{1}{2}\langle d|M|d\rangle >\delta \frac{2}{3}\langle d|P|d\rangle . \end{array}$$

Thus, for any $t>N_{0}$, it follows from Equations (17) and (18) that

(19) $$\begin{array}{c} D(\rho^{*}\left|\right|\rho^{t})\geq \delta D(p^{*}\left|\right|p^{t}). \end{array}$$

From Equation (12), we know

$$\begin{array}{cc} \; & \sum_{x}p_{x}^{*}\log\left(\frac{p_{x}^{t+1}}{p_{x}^{t}}\right)\geq D(\rho^{*}\left|\right|\rho^{t})\\ \Rightarrow & D(p^{*}\left|\right|p^{t+1})\leq D(p^{*}\left|\right|p^{t})-D(\rho^{*}\left|\right|\rho^{t}), \end{array}$$

combined with Equation (19), we have

(20) $$\begin{array}{cc} \; & D(p^{*}\left|\right|p^{t+1})\leq D(p^{*}\left|\right|p^{t})-\delta D(p^{*}\left|\right|p^{t})=\left(1-\delta \right)D(p^{*}\left|\right|p^{t})\\ \; & ⟹D(p^{*}\left|\right|p^{t}{)\leq \left(1-\delta \right)}^{t-N_{0}}D(p^{*}\left|\right|p^{N_{0}}) \end{array}$$

for any $t>N_{0}$. □

**Remark** **2.** (Complexity). Denote n,m as the size of input alphabet and output state dimension, respectively. A closer look at Algorithm 1 reveals that, for each iteration, a matrix logarithm $\log\rho^{t}$ needs to be calculated, and the rest are just multiplication of matrices and multiplication of numbers. The matrix logarithm can be done with complexity $O(m^{3})$ [16], thus, by Theorem 4 and Equation (11), the complexity to reach ε-close to the true capacity using Algorithm 1 is $O(\frac{m^{3}\log n\log\varepsilon }{\varepsilon })$. With extra condition of the channel ${\left\{\rho_{x}\right\}}_{x\in X}$, which is Assumption 1, the complexity to reach an ε-close solution (i.e., $D(p^{*}||p^{t})<\varepsilon$) using Algorithm 1 is $O(m^{3}\log\varepsilon \log_{\left(1-\delta \right)}\frac{\varepsilon }{D(p^{*}||p^{N_{0}})})$. Usually, we do not need ε to be too small (no smaller than $10^{-6}$), thus, in either case, the complexity is better than $O(\frac{\left(n\vee m\right)m^{3}{\left(\log n\right)}^{1/2}}{\varepsilon })$ in [6] when n∨m is big, where n∨m=max{n,m}.

## 4. Numerical Experiments on BA Algorithm

We only performed experiments on BA algorithm with no input constraint (BA algorithm with input constraint is some combination of BA algorithm with no input constraint.) We studied the relations between iteration complexity and n,m (i.e., the input size and output dimension) when the algorithm reaches certain accuracy. Since we do not know the true capacity of a certain channel, we used the following theorem to bound the error of the algorithm.

**Theorem** **8.** *With the iteration procedure in the BA Algorithm 1, $\max_{x}\{D(\rho_{x}\left|\right|\rho^{t})-\lambda s_{x}\}$ converges to F(λ) from above.*

**Proof.**  Following from Algorithm 1, Corollary 1, and Theorem 3, we have

$$\begin{array}{c} \lim_{t\to \infty }\frac{p_{x}^{t+1}}{p_{x}^{t}}=\exp[D(\rho_{x}\left|\right|\rho^{*})-\lambda s_{x}-F\left(\lambda \right)], \end{array}$$

where $\rho^{*}=\sum_{x}p_{x}^{*}\rho_{x}$, $p^{*}$ is an optimal distribution. The limit above is 1 if $p_{x}^{*}>0$ and does not exceed 1 if $p_{x}^{*}=0$. Thus,

$$\begin{array}{c} D(\rho_{x}\left|\right|\rho^{*})-\lambda s_{x}\leq F\left(\lambda \right) \end{array}$$

for every x∈X, with equality if $p_{x}^{*}>0$. This proves

$$\begin{array}{c} \max_{x}\{D(\rho_{x}\left|\right|\rho^{t})-\lambda s_{x}\}\to F\left(\lambda \right). \end{array}$$

For any $p^{t}$ and any optimal distribution $p^{*}$, we have

$$\begin{array}{cc} \max_{x} & [D(\rho_{x}\left|\right|\rho^{t})-\lambda s_{x}]\geq \sum_{x}p_{x}^{*}[D(\rho_{x}\left|\right|\rho^{t})-\lambda s_{x}]\\ \; & =\sum_{x}p_{x}^{*}D(\rho_{x}\left|\right|\rho^{*})+D(\rho^{*}\left|\right|\rho^{t})-\lambda s^{T}p^{*}\\ =F\left(\lambda \right)+D(\rho^{*}||\rho^{t})\geq F\left(\lambda \right). \end{array}$$

The first equality requires some calculation and the second equality follows since $p^{*}$ is an optimal distribution. This means $\max_{x}\{D(\rho_{x}\left|\right|\rho^{t})-\lambda s_{x}\}$ converges to F(λ) from above. □

Thus, our accuracy criterion was: for a given classical-quantum channel, we ran the BA algorithm (with no input constraint), until $[\max_{x}\{D(\rho_{x}\left|\right|\rho^{t})\}-f\left(p^{t},p^{t}\right)]$ was less than $10^{-k}$, and recorded the number of iteration. At this time, the accuracy was of order $10^{-(k+1)}$ at most since $\max_{x}\{D(\rho_{x}\left|\right|\rho^{t})\}$ and $f(p^{t},p^{t})]$ converged to the true capacity from above and below, respectively.

**Figure 1:**
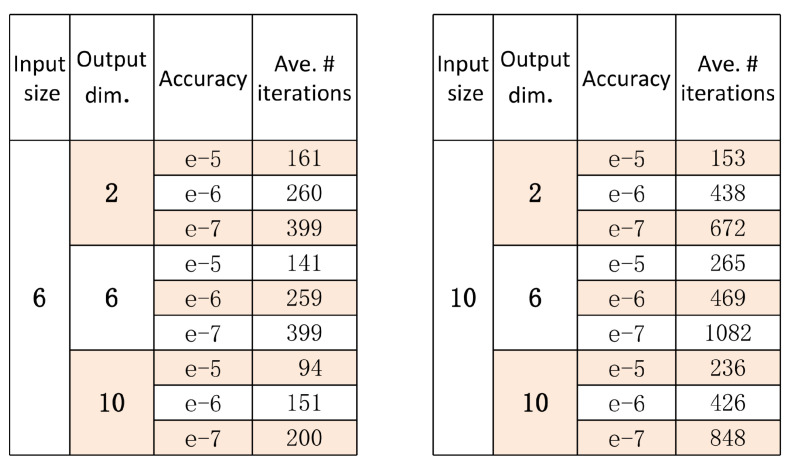
The number of iterations needed to reach certain accuracy.

We performed the following numerical experiments: for given values of input size *n*, output dimension *m* and accuracy, we generated 200 classical-quantum channels randomly, recorded the numbers of iterations, and then calculated the average number of iterations of these 200 experiments. The results are shown in Figure 1. Note that the accuracy $10^{-k}$ in Figure 1 means we ran the BA algorithm until $[\max_{x}\{D(\rho_{x}\left|\right|\rho^{t})\}-f\left(p^{t},p^{t}\right)]$ was less than $10^{-k}$, and the error between the true capacity and the computed value was of order $10^{-(k+1)}$ at most.

We can see in Figure 1 that the iteration complexity scales better as accuracy and input dimension increase. We can also see for given input size *n* and accuracy, the output dimension has vary little influence on iteration complexity, which means the iteration complexity also scales better as the output dimension *m* increases. Compared with our theoretical analysis of iteration complexity in Theorem 4: to reach ϵ accuracy, we needed $\frac{\log n}{\epsilon }$ iterations; the numerical experiments showed that the number of iterations was far smaller than $\frac{logn}{\epsilon }$ to reach ϵ accuracy, whether the output quantum states were independent or not (cases in (n,m)=(6,2),(10,2)). The reason for this is that the inequalities in the proof of Theorem 4 are quite loose. Thus, Theorem 4 only provides a very loose upper bound on iteration complexity. We can also guess that maybe the relation in Equation (20) holds generally and we just cannot prove it yet.

**Figure 2:**
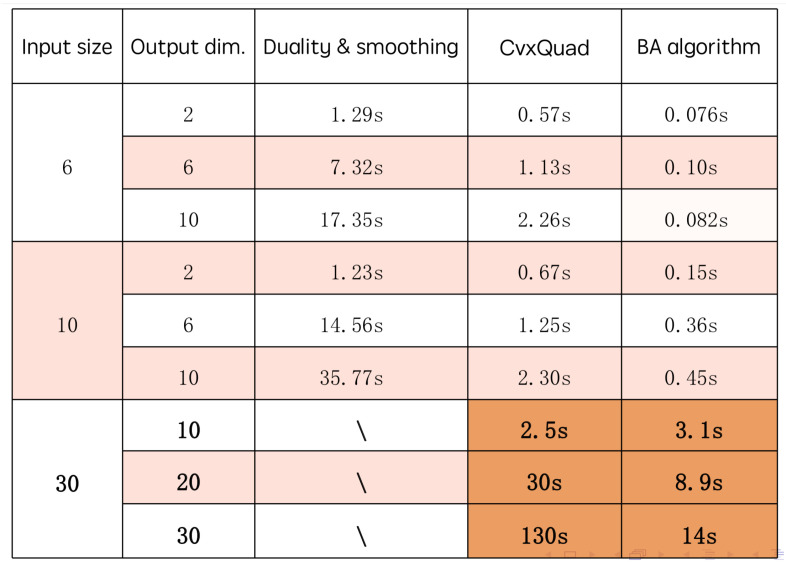
The caparison of the running time of three methods.

Next, we needed to see the running time of the BA algorithm. There were three methods to compute the classical-quantum channel capacity: BA algorithm, the duality and smoothing technique [6], and the method created by Hamza Fawzi et al. [17]. In [17], a Matlab code package called CvxQuad is provided which accurately approximates the relative entropy function via semidefinite programming so that many quantum capacity quantities can be computed using a convex optimization tool called CVX. Here, we compared the running time of the above three methods. For different input size *n* and output dimension *m*, we generated a classical-quantum channel randomly and computed the channel capacity using the above three methods and then recorded the running time of each method. The results are shown in Figure 2.

In Figure 2, we can see the BA algorithm was the fastest method. The duality and smoothing method was rather slow and we did not record the running time of the duality and smoothing method when n=30 because it took too long. We can also notice that the running time of the CvxQuad method was extremely sensitive to the output dimension, which is not a surprise because CVX is a second-order method. Thus, our BA algorithm was significantly faster than the other two methods when *n* and *m* became big.

## 5. An Approximate Solution of *p* in Binary Two Dimensional Case

In this section, we provide an approximate optimal input distribution for the case of the input size and output dimension are both 2:

$$\begin{array}{c} \left\{p_{1}:\rho_{1};p_{2}:\rho_{2}\right\},\;p_{1}+p_{2}=1,\rho_{1},\rho_{2}\in D^{2}. \end{array}$$

### 5.1. Use Bloch Sphere to Get an Approximate Solution

**Figure 3:**
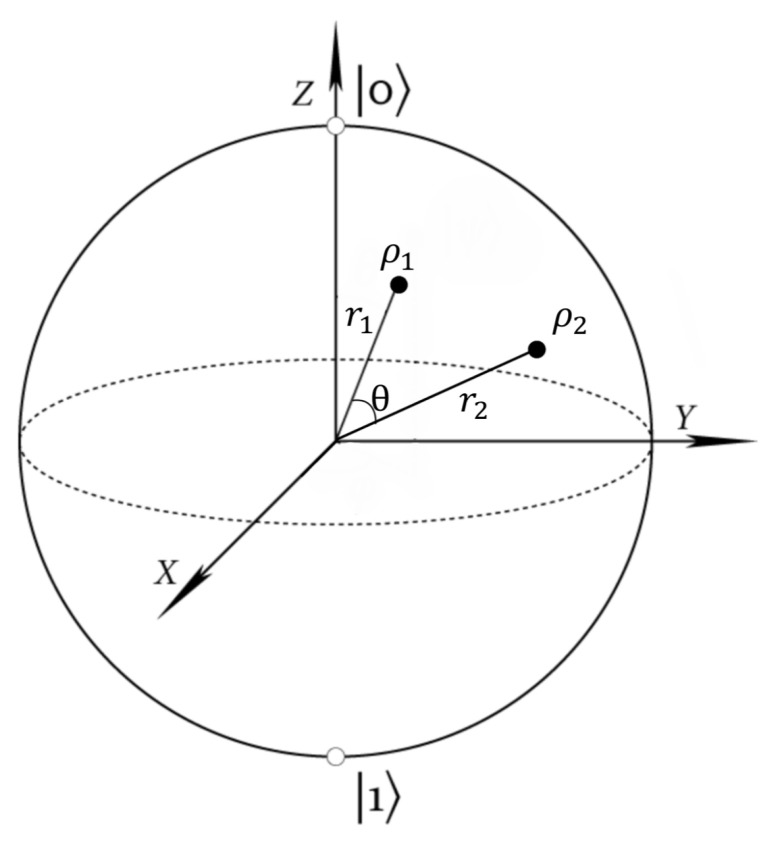
Bloch sphere.

Any two-dimensional density matrix can be represented as a point in the Bloch sphere [5], as shown in the following:

Any density matrix can be represented as a vector in the Bloch sphere starting from the origin. Suppose $\rho_{1},\rho_{2}$ can be represented as $r_{1},r_{2}$ respectively, as shown in Figure 3; then, the two eigenvalues would be $0.5\pm r_{1}/2$ and $0.5\pm r_{2}/2$, respectively. Extending $r_{1}$, we get two intersections on the surface of the Bloch sphere; then, these two intersections represent the two eigenvectors of $\rho_{1}$ (the points on the surface of the sphere represent pure state and the interior points represent mixed states). A probabilistic combination of $\rho_{1},\rho_{2}$ can be represented as $p_{1}\rho_{1}+p_{2}\rho_{2}=p_{1}r_{1}+p_{2}r_{2}$ ([5] Exercise 4.4.13). Any point on the surface of Bloch sphere can be represented as

$$\begin{array}{c} \cos\frac{\alpha }{2}|0\rangle +\sin\frac{\alpha }{2}e^{i\phi }|1\rangle , \end{array}$$

where α is the angle to the *Z*-axis and ϕ is the angle of the *X*-axis to the projection of the point on the *X*–*Y* plane.

By symmetry, it is obvious that the Holevo quantity is only related to $r_{1},r_{2},\theta ,p_{1}$, where θ is the angle between $r_{1},r_{2}$. One interesting result is that the angle θ has very little influence on $p^{*}$, where $p^{*}$ is the optimal distribution that maximizes Holevo quantity. If we know $\lambda_{1},\lambda_{2}$ (the bigger eigenvalues of $\rho_{1},\rho_{2}$, respectively), θ and $p_{1}$, then the Holevo quantity can be written as

(21) $$\begin{array}{c} \chi \left(\lambda_{1},\lambda_{2},\theta ,p_{1}\right)=S(\frac{1}{2}+\frac{1}{2}\left|\right|p_{1}r_{1}+\left(1-p_{1}\right)r_{2}{\left|\right|}_{2})-\left[p_{1}S\left(\frac{1}{2}+\frac{1}{2}r_{1}\right)+\left(1-p_{1}\right)S\left(\frac{1}{2}+\frac{1}{2}r_{2}\right)\right], \end{array}$$

where S(·) is the binary entropy (S(x)=−(xlogx+(1−x)log(1−x)) and $r_{i}=2\lambda_{i}-1$.

Using Cosine Theorem to calculate $\left|\right|p_{1}r_{1}+\left(1-p_{1}\right)r_{2}{\left|\right|}_{2})$, the gradient of χ with respect to $p_{1}$ can be calculated directly, denoted as

$$\begin{array}{c} \nabla_{p_{1}}\chi \left(\lambda_{1},\lambda_{2},\theta ,p_{1}\right). \end{array}$$

If we can find a $p_{1}$ such that $\nabla_{p_{1}}\chi \left(\lambda_{1},\lambda_{2},\theta ,p_{1}\right)=0$, then this $p_{1}$ is the optimal solution (because $\chi (\lambda_{1},\lambda_{2},\theta ,p_{1})$ is concave in $p_{1}$). However, we cannot solve the equation $\nabla_{p_{1}}\chi \left(\lambda_{1},\lambda_{2},\theta ,p_{1}\right)=0$ with respect to $p_{1}$ when θ≠0. Now that θ has little influence on $p^{*}$, let θ=0 (this is actually the classical case), and let

$$\begin{array}{c} \nabla_{p_{1}}\chi \left(\lambda_{1},\lambda_{2},\theta =0,p_{1}\right)=0, \end{array}$$

the above equation is easy to solve and we get a solution ${\hat{p}}_{1}$:

(22) $$\begin{array}{c} {\hat{p}}_{1}=\frac{\frac{1-c}{1+c}-r_{2}}{r_{1}-r_{2}},\;\;\mathrm{where}\;c=2^{\frac{S\left(\lambda_{1}\right)-S\left(\lambda_{2}\right)}{(r_{1}-r_{2})/2}}, \end{array}$$

where we assume $r_{1}\neq r_{2}$. (It can be easily seen from the Bloch sphere that if $r_{1}=r_{2}$, the optimal distribution would be $\left\{\frac{1}{2},\frac{1}{2}\right\}$.)

This ${\hat{p}}_{1}$ can be used as an approximate optimal solution for all θ∈[0,π]. Next, we need numerical experiments to see how accurate ${\hat{p}}_{1}$ is.

### 5.2. Numerical Experiments on the Approximated Solution ${\hat{p}}_{1}$

It is obvious that the maximum of Holevo quantity only depends on $r_{1},r_{2}$ and θ, thus, without loss of generality, we let $\rho_{1}$ be on the *Z*-axis and $\rho_{2}$ be on the *X*–*Z* plane:

$$\begin{array}{cc} \; & \rho_{1}=\lambda_{1}\left|0\rangle \langle 0\right|+\left(1-\lambda_{1}\right)|1\rangle \langle 1|;\\ \; & \rho_{2}=\lambda_{2}|\psi_{0}\rangle \langle \psi_{0}|+\left(1-\lambda_{2}\right)|\psi_{1}\rangle \langle \psi_{1}|, \end{array}$$

where

$$\begin{array}{cc} \; & |\psi_{0}\rangle =\cos\frac{\theta }{2}|0\rangle +\sin\frac{\theta }{2}|1\rangle ;\\ \; & |\psi_{1}\rangle =-\sin\frac{\theta }{2}|0\rangle +\cos\frac{\theta }{2}|1\rangle , \end{array}$$

which means the angle between $\rho_{1}$ and $\rho_{2}$ (i.e., $r_{1}$ and $r_{2}$) is θ [5].

In the numerical experiments, we let $\lambda_{1},\lambda_{2}$ range from 0.5 to 1, and θ range from 0 to π. For each value of $\left(\lambda_{1},\lambda_{2},\theta \right)$, we substituted $\left(\lambda_{1},\lambda_{2}\right)$ into Equation (22) to compute ${\hat{p}}_{1}$. Then, we substituted $\left(\lambda_{1},\lambda_{2},\theta ,{\hat{p}}_{1}\right)$ into Equation (21) to get the approximate maximum of Holevo quantity over $p_{1}$: $\chi (\lambda_{1},\lambda_{2},\theta ,{\hat{p}}_{1})$. To see how accurate this approximate maximum is, we need a BA algorithm to provide an accurate maximum. The termination criterion for the iteration process of BA algorithm is stopping when $[\max_{x}\{D(\rho_{x}\left|\right|\rho^{t})\}-f\left(p^{t},p^{t}\right)]$ is less than $10^{-6}$; then, the BA algorithm outputs a value of Holevo quantity $\chi_{BA}\left(\lambda_{1},\lambda_{2},\theta \right)$. We can compute the error of $\chi (\lambda_{1},\lambda_{2},\theta ,{\hat{p}}_{1})$ then take the maximum over θ∈[0,π]

$$\begin{array}{c} \mathrm{Error}\left(\lambda_{1},\lambda_{2}\right)=\max_{\theta \in [0,\pi ]}\left|\chi \left(\lambda_{1},\lambda_{2},\theta ,{\hat{p}}_{1}\right)-\chi_{BA}\left(\lambda_{1},\lambda_{2},\theta \right)\right|. \end{array}$$

**Figure 4:**
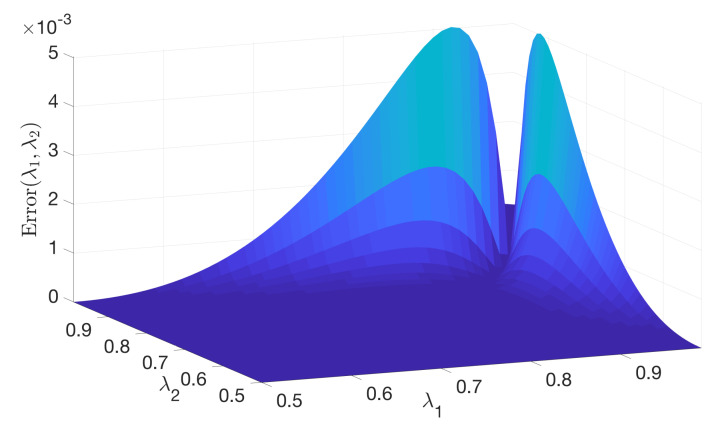
Error of the approximated method.

Figure 4 is the numerical result, which is a plot of $\left(\lambda_{1},\lambda_{2},\mathrm{Error}\left(\lambda_{1},\lambda_{2}\right)\right)$.

In Figure 4, we can see that, if $\lambda_{1},\lambda_{2}$ are not “too big", the error can be upper bounded by $10^{-3}$. To see this more directly, we take the maximum of $\mathrm{Error}(\lambda_{1},\lambda_{2})$ for different ranges of $\lambda_{1},\lambda_{2}$:

$$\begin{array}{c} \max_{\lambda_{1},\lambda_{2}\in \left[0.5,R\right]}\mathrm{Error}\left(\lambda_{1},\lambda_{2}\right). \end{array}$$

**Figure 5:**
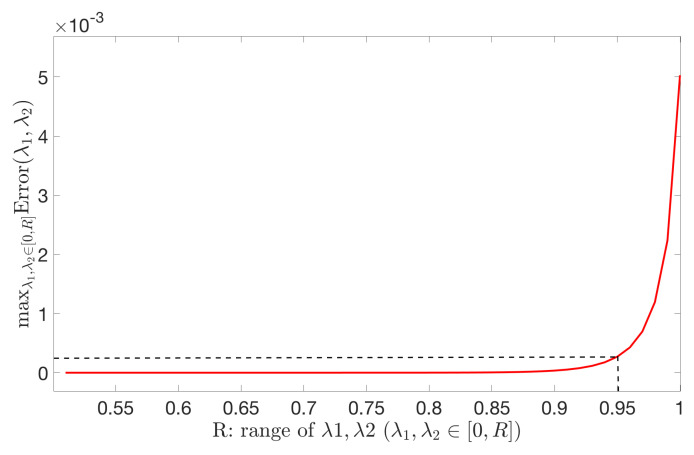
Error of the approximate method.

Figure 5 is a plot of $\left(R,\max_{\lambda_{1},\lambda_{2}\in \left[0.5,R\right]}\mathrm{Error}\left(\lambda_{1},\lambda_{2}\right)\right)$.

In Figure 5, we can see that, if $\lambda_{1},\lambda_{2}<0.95$, the error of approximate maximum of Holevo quantity can be upper bounded by $3\times 10^{-4}$. Thus, we can conclude that, when the bigger eigenvalues of $\rho_{1},\rho_{2}$ are not too big (no bigger than 0.95), Equation (22) can make the error of the maximum of Holevo quantity smaller than $3\times 10^{-4}$.

The approximate solution is an interesting phenomenon. The reason the angle θ has such little influence on the maximum of Holevo quantity is unclear.

## 6. Discussion

In this paper, we provide an algorithm which computes the capacity of classical-quantum channel. We analyzed the speed of convergence theoretically and numerical experiments showed that our algorithm outperforms the existing methods [6,17]. We also provide an approximated method to compute the capacity of binary two-dimensional classical-quantum channel, which shows high accuracy. As mentioned in the Introduction, for a general quantum-quantum channel, maximizing Holevo quantity with respect to both the input distribution and output quantum state is NP-complete and this is not a convex optimization problem because Holevo quantity is concave with respect to input distribution and convex with respect to output quantum states. Thus, it remains open whether there exists an efficient algorithm to solve this problem. However, for classical-quantum channel, Holevo quantity has an upper bound, thus one future work would be to maximize Holevo quantity with respect to output states with given input distribution. It remains open if alternating optimization algorithms, in particular of Blahut–Arimoto type, can also be given for other optimization problems in terms of quantum entropy.

The authors declare no conflict of interest.
